# Reasons and clinical outcomes of antipsychotic treatment switch in outpatients with schizophrenia in real-life clinical settings: the ETOS observational study

**DOI:** 10.1186/1744-859X-12-42

**Published:** 2013-12-20

**Authors:** Andreas Roussidis, Christina Kalkavoura, Dimos Dimelis, Afroditi Theodorou, Ina Ioannidou, Eleytherios Mellos, Triantafyllia Mylonaki, Areti Spyropoulou, Andreas Yfantis

**Affiliations:** 1 AstraZeneca Greece, Athens 15125, Greece; 2 Psychiatric Department, Sismanoglio General Hospital, Athens 15126, Greece; 3 Private Practice, Thessaloniki 54623, Greece; 4 Private Practice, Athens 11528, Greece; 5 Private Practice, Nafplio 21100, Greece; 6 Aiginition Hospital, Athens 11528, Greece; 7 Center for Mental Health, Kalamata 24100, Greece

**Keywords:** Schizophrenia, Clinical outcome, Antipsychotic, Switch, Monotherapy

## Abstract

**Background:**

Patients under antipsychotic treatment for schizophrenia commonly exhibit poor adherence to treatment, high rates of treatment discontinuation, and frequent treatment changes. The ETOS study aimed to identify the reasons leading physicians to decide to switch antipsychotic treatment in outpatients with schizophrenia and to evaluate the outcome of this switch.

**Methods:**

ETOS was an observational 18-week (four visits) study in outpatients 18 to 65 years old, diagnosed with schizophrenia according to Diagnostic and Statistical Manual of Mental Disorders - 4th edition criteria at least 6 months prior to enrolment, who were initiated on a new antipsychotic monotherapy treatment within the 2 weeks prior to enrollment. A total of 574 patients were recruited by 87 hospital- and office-based physicians. Ethical approval was obtained prior to study initiation (NCT00999895).

**Results:**

The final analysis included 568 patients, 39.0 ± 11.2 years old with mean disease duration of 11.7 years. The male-to-female ratio was 53:47. The main reason for switching antipsychotic treatment was lack of tolerability (*n* = 369, 65.0%), followed by lack of efficacy (*n* = 249, 43.8%). Following treatment switch, 87.9% of patients (*n* = 499) showed meaningful clinical benefit by achieving a Clinical Global Impression-Clinical Benefit score of ≤4 at the final visit. By the end of the study, total Positive and Negative Syndrome Scale, Clinical Global Impression-Improvement, Clinical Global Impression-Severity, and Simpson-Angus Scale scores demonstrated significant mean decreases of 31.69, 0.70, 1.14, and 11.30, respectively (all *p* < 0.0001). Treatment adherence remarkably improved.

**Conclusion:**

In the ETOS study, switch of antipsychotic monotherapy for reasons relating to lack of efficacy and/or tolerability was associated with significantly improved clinical benefit and significant increase of patients' adherence to treatment.

## Background

Schizophrenia is a chronic, severely disabling psychiatric illness with a lifetime risk ranging from 0.2% to 0.7% and an incidence of 15.2 per 100,000 per year [1,2]. Treating schizophrenia is usually a lifelong process that poses an enormous disease burden on the patient and the caregiver and a great challenge for the physician. Maintenance therapy and adherence to treatment are crucial factors for the long-term clinical management of schizophrenia and are key determinants for good prognosis [3-6].

Schizophrenic patients under antipsychotic treatment have been shown to exhibit poor adherence and high rates of treatment discontinuation, often within the first year of treatment commencement [7-11]. It has been shown that almost half of the patients with schizophrenia or schizoaffective disorder on antipsychotic medication take less than 70% of the prescribed doses [12]. Partial or no adherence to treatment has been associated with increased risk of relapse, psychiatric hospitalization, attempted suicide, and clinical and functional deterioration [13-15]. Poor treatment adherence and high discontinuation rates often lead to frequent treatment changes, as it is generally accepted that subjects who are not responding to an agent belonging to a particular class of psychotropic drugs or who experience adverse events may show a better response to another agent of the same or other therapeutic class [16,17].

Switching to improve efficacy and/or tolerability has been studied in several clinical trials. A significant improvement in the Positive and Negative Syndrome Scale (PANSS) scores as well as in patients' metabolic profile has been observed by switching from conventional antipsychotics, olanzapine, or risperidone to ziprasidone over a 6-week clinical study [18]. Cognitive function has been shown to improve by switching from conventional or atypical antipsychotics to ziprasidone [19] or to olanzapine [20]. Ganesan and colleagues have shown that switching from other antipsychotics to quetiapine XR is associated with an improved efficacy and tolerability profile [21]. Furthermore, switching to quetiapine from typical or atypical antipsychotics has been found to significantly reduce extrapyramidal symptoms [22], and switching to quetiapine from another atypical agent has been proposed in cases of new-onset tardive dyskinesia [23,24]. Nonetheless, the question regarding whether a switch in antipsychotic treatment improves the patients' outcome still remains unanswered. Improvement in patient outcome is probably related to the therapeutic choice made each time [17].

Atypical antipsychotics are considered to be better tolerated than typical antipsychotic agents and are currently the mainstay of schizophrenia treatment. However, atypical antipsychotics have been associated with long-term adverse effects on patients' weight, serum glucose levels, and serum lipids [25-28]. In order to develop a patient-individualized switching strategy, decision making should be based on key patient illness characteristics, specifically on the patient's symptomatology, comorbidities, and side effects experienced with previous antipsychotics [16].

The ETOS study aimed to identify the reasons leading physicians to change the single antipsychotic treatment of outpatients with schizophrenia to another single antipsychotic agent and to evaluate the outcome of this switch.

## Methods

### Study design

ETOS was an open-label, prospective, observational study that enrolled 574 outpatients with schizophrenia, who required a change in their primary antipsychotic medication for any reason according to their physician's discretion. The study was conducted between October 2009 and September 2010 in 87 sites all over Greece; ten of these sites were hospital-based, while the remaining 77 were private office-based practices.

The study was conducted in accordance with the Declaration of Helsinki and approved both by the Scientific Committee/Administrative Council (IRBs) of the participating hospitals and the Greek National Organization of Medicines. All aspects of treatment and care of patients were determined by the treating physicians. Ethical approval was obtained prior to study initiation (NCT00999895).

### Study population

Male or female outpatients were included in the study if they (1) were aged between 18 to 65 years, (2) had a diagnosis of schizophrenia according to Diagnostic and Statistical Manual of Mental Disorders - 4th edition (DSM-IV) at least 6 months prior to enrollment, and (3) were initiated on a new antipsychotic monotherapy treatment within the preceding 2 weeks. All patients were receiving antipsychotic monotherapy before switching to a new antipsychotic treatment. The provision by the patient of a written informed consent was a prerequisite for entering the study.

Exclusion criteria were as follows: (1) diagnosis of any other psychiatric condition (except from schizophrenia) as per DSM-IV Axis Ι, concomitant organic mental disorder or mental retardation, (2) substance abuse or dependence (with the exception of nicotine dependence) as defined by DSM-IV criteria and not in full remission, (3) pregnancy or breastfeeding, and (4) participation in another clinical study.

### Patient assessments

The study was conducted in the real-world clinical practice. Patients were observed by their treating physicians during four scheduled visits: on day 0 (baseline), week 6 (visit 2), week 12 (visit 3), and week 18 (final visit), over a period of 18 weeks. Sociodemographic and baseline characteristics, including age, anthropometric, and medical history, were recorded during the baseline (day 0) visit. Laboratory results, when available, including those that led to the decision of treatment switch and concomitant medications were recorded throughout the study. Clinical benefit from treatment switching which was the primary study objective was assessed with the Clinical Global Impression-Clinical Benefit (CGI-CB) scale by calculating the percentage of subjects achieving a score of ≤4 (1 indicates the greatest improvement) at the end of the study.

The study also aimed to capture the detailed reasons which led the physicians to the decision of switching antipsychotic treatment, as well as the therapeutic management options. Thus, during the baseline visit, physicians recorded the reasons which led them to switch the patients' previous antipsychotic monotherapy as well as the patients' previous and current antipsychotic medication. In patients who demonstrated lack of efficacy as the reason for treatment switching, the Positive and Negative Syndrome Scale (PANSS) was used to evaluate the effect of the new treatment. On the other hand, in patients who demonstrated lack of tolerability, the Simpson-Angus Scale (SAS) scores were recorded in cases where the reason for switching was extrapyramidal symptoms, while measurements of body weight, were employed for those switching due to problems with their body weight. Furthermore, when the reason for switching was abnormal laboratory values, the respective variable, i.e., serum glucose levels, prolactin levels, and serum lipids were evaluated. Standardized efficacy parameters such as the CGI-Improvement (CGI-I) and CGI-Severity (CGI-S) were evaluated for all patients. Compliance to the new treatment was also evaluated for all patients by the use of Brief Adherence Rating Scale (BARS). The aforementioned secondary variables were monitored at the baseline visit (except for the CGI-I score), the two follow-up visits and the final visit. Due to the non-interventional nature a comprehensive safety assessment was not conducted.

### Statistical analysis

Statistical analysis was performed in the study population attending all follow-up visits (Per Protocol Population-PP) using the statistical software SPSS 17.0. The analysis of data was based mainly on descriptive statistical methods. Continuous variables are presented as means of measures of central tendency and dispersion. Categorical variables are presented as frequency distribution tables. The relationship between categorical variables is illustrated using contingency tables. The statistical significance of the changes in the evaluation scales and laboratory measurements between the first and final visit was assessed by means of a paired *t* test. The 95% confidence intervals were calculated regarding the estimation of the primary endpoint.

## Results

### Patients’ characteristics

Of the 574 patients initially enrolled, 568 patients (98.95%) comprised the Per Protocol (PP) population used in the present analysis. The six patients not included in the PP population were either lost to follow-up or did not attend all study visits. Specifically, of the 574 patients attending the first visit, 571 attended the second visit, 569 the third visit, and 569 the final visit (with one patient returning for the final visit after missing visits 2 and 3).

Baseline sociodemographic and clinical characteristics for the study population are presented in Table 1. The study population had a mean age of 39 ± 11.2 years with a male-to-female ratio of 53:47. The mean time since schizophrenia diagnosis was 11.7 years. The majority of the study population was urban residents (70.1%), married or living with a spouse and/or children or relatives (83.5%), at least high school graduates (66.2%), and unemployed (61.2%). The most common concomitant diseases (>1%) were psychiatric disorders - other than schizophrenia - (32.7%), neurological comorbidities (12.7%), vascular disorders (5.8%), and metabolic and nutritional disorders (5.1%). The majority of the patients (*n* = 371, 65.3%) were receiving at least one concomitant medication during the 18-week study period.

**Table 1:** Baseline demographic and clinical characteristics

	**Number**	**Percent**	**Mean ± SD (range)**
Gender			
Total	568	100.0	
Male	301	53.0	
Female	267	47.0	
Educational level			
Elementary school	71	12.5	
Junior high school	121	21.3	
High school	217	38.3	
Technical institution	90	15.9	
University	68	12.0	
Living conditions			
Lives alone	86	15.1	
Lives with spouse	101	17.8	
Lives with children or relatives others	372	65.5	
Nursing home, institution	8	1.4	
Lives with wife and children	1	0.2	
Age (years)			39.0 ± 11.2 (18.0 to 65.0)
BMI (kg/m^2^)			27.6 ± 4.7 (17.7 to 50.8)
Time since diagnosis (year)			11.7 ± 12.3 (0.5 to 41.0)
PANSS	249		92.9 ± 28.2
CGI-S	568		4.1 ± 1.1
SAS	111		14.5 ± 9.6
BARS	568		86.1 ± 18.2

BMI, body mass index; PANSS, Positive and Negative Syndrome Scale; CGI-S, Clinical Global Impression-Severity; SAS, Simpson-Angus Scale; BARS, Brief Adherence Rating Scale.

### Reasons for switching antipsychotic treatment

The main reason for leading physicians' to switching antipsychotic treatment in schizophrenic outpatients was lack of tolerability (*n* = 369, 65.0%), followed by lack of efficacy (*n* = 249, 43.8%). Notably, 8.8% (*n* = 50) patients were switched due to lack of both tolerability and efficacy. The two major tolerability reasons were weight gain (40.4%) and extrapyramidal symptoms (30.1%) (Table 2). Patients who changed treatment for tolerability reasons (*n* = 369) were mainly switched from olanzapine (37.4%) and risperidone (24.7%). Patients who switched due to lack of efficacy (*n* = 249) were mainly (>10%) switched from aripiprazole (22.1%), risperidone (21.3%), olanzapine (16.5%), and ziprasidone (12.9%).

**Table 2:** Reasons for switching antipsychotic treatment

**Reason**	**Number**	**Percent**
Lack of efficacy		
Alone	199	35.0
In combination with lack of tolerability	50	8.8
Total	249	43.8
Lack of tolerability		
Alone	319	56.2
In combination with lack of efficacy	50	8.8
Total	369	65.0
Lack of tolerability, analytically (*n* = 369)*		
Weight gain	149	40.4
Extrapyramidal symptoms	111	30.1
Lack of tolerance	42	11.4
Hyperprolactinaemia	39	10.6
Hyperlipidaemia and/or glucose increase	24	6.5
Stress/insomnia/anxiety/akathisia	16	4.3
Sleepiness/drowsiness	12	3.2
Gynecological dysfunctions	6	1.6
Sexual disorders	6	1.6
Other adverse events	10	2.7

Asterisk ‘*’ denotes more than one of these reasons may have been selected.

### Current antipsychotic treatment

Patients who were switched from their previous antipsychotic treatment due to efficacy reasons were mainly switched to quetiapine (50.2%), risperidone (8.8%), aripiprazole (7.6%), olanzapine (7.6%), amisulpride (7.2%), and paliperidone (6.4%). Patients who were switched due to tolerability reasons were mainly switched to quetiapine (58.5%), aripiprazole (10.8%), olanzapine (9.8%), paliperidone (6.2%), and ziprasidone (5.4%).

### Clinical benefit from switching to another antipsychotic monotherapy

**Figure 1:**
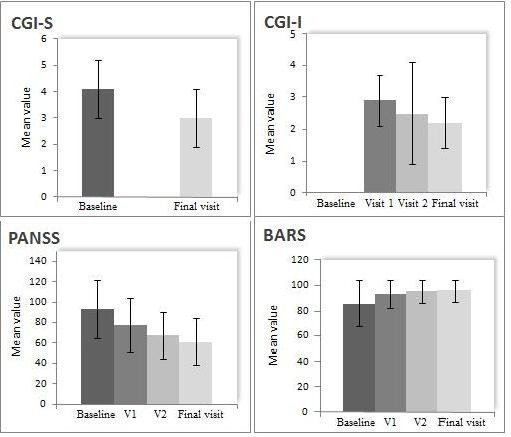
**Total mean values in CGI-S, CGI-I, PANSS, and BARS at baseline, visit 1, visit 2, and final visit.** After 18 weeks of treatment. Results for CGI-S, CGI-I, and BARS refer to the total population. PANSS refers only for these patients who demonstrated lack of efficacy as the reason for treatment switching.

The physician's strategy to manage their schizophrenic patients who presented with suboptimal efficacy or tolerability by switching the antipsychotic treatment resulted in a meaningful clinical benefit for 87.9% (95% CI, 84.9 to 90.4) of patients (*n* = 499) as assessed by achieving a CGI-CB score of ≤ 4 at the final visit. Clinical benefit, i.e., CGI-CB ≤4, was achieved by 86.9%, 89.0%, and 84.0%, respectively, of patients who switched therapy for efficacy, tolerability, or for both reasons. Additionally, by the end of the study, the mean CGI-I and CGI-S scores demonstrated a significant mean decrease from baseline of 0.71 (95% CI, 0.76 to 0.64; *p* < 0.001) and 1.14 (95% CI, -1.22 to -1.05; *p* < 0.001), respectively (Figure 1).

### Efficacy as reason for switching

The patients whose physicians decided to change their therapeutic management due to efficacy reasons had a mean total PANSS baseline score of 92.9 (± 28.2) (*n* = 249). At the end of the follow-up period, 18 weeks following the change of their treatment, the total PANSS scores were significantly improved showing a mean decrease from baseline of 31.69, (95% CI, -34.42 to -28.96; *p* < 0.0001). The mean values of PANSS at baseline are presented in Table 1, while an overview of the aforementioned efficacy measures at each visit is shown in Figure 1.

### Tolerability assessments

All patients who switched due to extrapyramidal symptoms were evaluated with SAS (*n* = 111) at baseline and subsequent study visits and presented a significant improvement, with a mean decrease in SAS score of 11.30 (95% CI, -13.09 to -9.51; *p* < 0.0001). Following treatment switch, body weight, glucose, total cholesterol, and prolactin levels showed significant decreases by the end of the study (Table 3). In particular, measurements in body weight were recorded for those patients (*n* = 149) experiencing clinically significant weight gain with their previous antipsychotic medication and showed a mean decrease of 6.85 kg, (95% CI, -7.75 to -5.96; *p* < 0.0001) by the end of the study. Prolactin levels were also significantly decreased by 62.3 ng/mL (95% CI, -76.7 to -47.9; *p* < 0.0001) in those patients who had switched due to hyperprolactinaemia (*n* = 39).

**Table 3:** Mean changes in weight, glucose, prolactin, and serum lipid levels from baseline to final visit

**Measurement**	**Number**	**Baseline, mean (SD)**	**Final visit, mean (SD)**	**Mean change**	***p *** **value**
Weight (kg)	149	89.6 (17.3)	82.8 (16.0)	-6.85	<0.0001
Glucose (mmol/L)	17	48.3 (69.4)	36.2 (49.8)	-12.08	0.022
Prolactin (ng/mL)	39	83.6 (48.7)	21.3 (10.8)	-62.3	<0.0001
Total cholesterol (mmol/L)	11	81.7 (108.0)	72.0 (96.2)	-9.73	0.047
LDL (mmol/L)	8	75.8 (92.2)	70.2 (84.4)	-5.52	0.445
HDL (mmol/L)	10	37.4 (17.9)	41.6 (17.7)	4.2	0.282
Triglycerides (mmol/L)	10	124 (NA)	92.4 (112.3)	-31.58	0.212

LDL, low-density lipoprotein; HDL, high-density lipoprotein; NA not applicable.

### Treatment adherence

Adherence to treatment was evaluated in the total study population using the BARS scores (Table 1, Figure 1). Treatment switch was accompanied by significant improvement in adherence, as shown by a mean change in BARS scores of 9.73 at the end of the study compared to baseline (95% CI, 8.38 to 11.08; *p* < 0.0001).

## Discussion

Since the introduction of second-generation antipsychotics in schizophrenia, there has been a change in the strategy of treatment - from inpatient treatment of the acute symptoms to outpatient maintenance treatment and improved quality of life. The golden standard of antipsychotic treatment should aim to achieve ‘clinical stabilization’ after achieving remission of symptoms in the acute phase, thus reducing the risk for progressive cognitive deterioration, functional disabilities, comorbidities, and poor quality of life [29,30]. It has been shown that lack of efficacy and tolerability, often associated with poor compliance, results in treatment discontinuation or treatment switch [8,31].

In our study, the switch to a second-generation antipsychotic monotherapy treatment led to a clinical benefit which was achieved within an 18-week period by the majority of the participants, irrespective of the reason(s) for switch. Furthermore, all efficacy measures - total PANSS, CGI-I, and CGI-S scores - were significantly improved, justifying the physicians’ choice of treatment. Treatment switch was well tolerated by the majority of the study participants; SAS scores as well as certain metabolic parameters, such as body weight, glucose, total cholesterol and prolactin levels, were significantly improved. Notably, upon the decision to switch, the safety profile of different antipsychotics must be taken into consideration, as antipsychotic medications are known to have varying degrees of adverse effects on body weight and can lead to increased risk for diabetes and dyslipidaemia [32]. Furthermore, our study population showed increased adherence to treatment, which was sustained throughout the 18 weeks of the study.

A major issue that should be considered when switching between atypical antipsychotics is the occurrence of side effects or withdrawal symptoms, many of which are attributed to receptor profiles and antimuscarinic or antihistaminic blockade [16]. In the ETOS study, tolerability was only assessed in those patients experiencing tolerability issues with their past medication. However, as treatment switch was accompanied by increased compliance in the majority of the study population, it can be postulated that patients switched to another antipsychotic due to efficacy reasons did not experience major side effects - i.e., extrapyramidal symptoms, weight gain, or metabolic disorders - or withdrawal symptoms, at least not to the extent that the possible side effects might have interfered with the patients' adherence to treatment. At the same time, patients that were switched due to tolerability reasons showed increased efficacy, a fact that may underline what has been previously suggested, i.e., reducing side effects improves tolerability and therefore may improve compliance, which in turn may be translated into enhanced efficacy [12,16].

Limitations of the study include its non-randomized nature, which may have introduced bias. Secondly, the fact that the majority of patients were switched to quetiapine makes it difficult to draw any conclusions from head-to-head drug comparisons, which therefore have been avoided; thirdly, physicians were not asked to register the reasons for selecting a particular antipsychotic. This lack of information prevents us from drawing firm conclusions on the safety-specific characteristics of the different antipsychotics that may guide physician decision making, especially in high-risk patients, such as dyslipidaemic patients.

In conclusion, when an antipsychotic treatment shows lack of efficacy or has tolerability issues, despite efforts for optimal dosing and control of the associated side effects, switching to another single antipsychotic agent may be efficacious, well tolerated, and may result in an increased adherence to treatment. Once the decision on switching therapy has been taken, the switching strategy must be tailored to the individual patient characteristics and environmental factors. The transition period is crucial, as side effects to the new agent may be transient and withdrawal symptoms may occur, and therefore, both the physician and the family/caregiver should actively support the patient encouraging adherence and persistence to treatment.

## Abbreviations

BARS: Brief adherence rating scale; CGI-CB: Clinical global impression-clinical benefit; CGI-Ι: Clinical global impression-improvement; CGI-S: Clinical global impression-severity; DSM-IV: Diagnostic and statistical manual of mental disorders - 4th edition; PANSS: Positive and negative syndrome scale; SAS: Simpson-angus scale.

## Competing interests

AR is an employee of AstraZeneca.

## Authors’ contributions

AR and CK contributed substantially to the design and analysis of the study, as well as to development and critical revision of the manuscript. DD, AT, II, EM, TM, AS, and AY have contributed substantially to the interpretation of the data and critical revision of the manuscript. All authors read and approved the final manuscript.
